# Combined photodynamic therapy and intravitreal bevacizumab as primary treatment for subretinal neovascularization associated with type 2 idiopathic macular telangiectasia

**DOI:** 10.4103/0301-4738.49406

**Published:** 2009

**Authors:** Pukhraj Rishi, Ekta Rishi, Daraius Shroff

**Affiliations:** Shri Bhagwan Mahavir Vitreoretinal Services, Medical and Vision Research Foundations, Sankara Nethralaya, 18, College Road, Chennai - 600 006, Tamil Nadu, India

Dear Editor,

The natural history of untreated subretinal neovascular membranes (SRNVM) associated with idiopathic macular telangiectasia (IMT) is generally poor with 81% of eyes in a series of 26 eyes having a final visual acuity of 20/200 or worse.[1] Photocoagulation and sub-retinal surgery for treatment of SRNVM associated with IMT have not shown good results.[2,3] Photodynamic therapy (PDT) and intravitreal bevacizumab have been used individually for treatment of such eyes;[4,5] Combination PDT and ranibizumab has also been reported.[6] However, there are no reports on combination PDT and intravitreal bevacizumab for such eyes.

**Figure 1:**
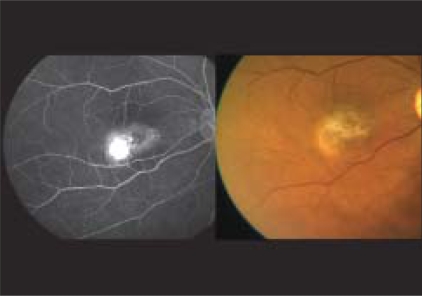
Color fundus picture (right) reveals perifoveal retinal opacification, refractile crystalline deposits and SRNVM temporal to the fovea along with ILM striae suggestive of proliferative Type 2 idiopathic macular telangiectasia. Fundus fluorescein angiography (left) reveals intense leakage from the SRNVM

**Figure 2:**
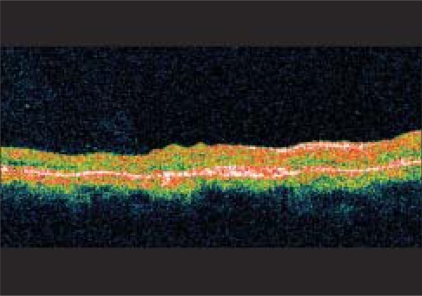
At presentation, optical coherence tomography reveals increased retinal thickening overlying the SRNVM with subretinal fluid (SRF)

**Figure 3:**
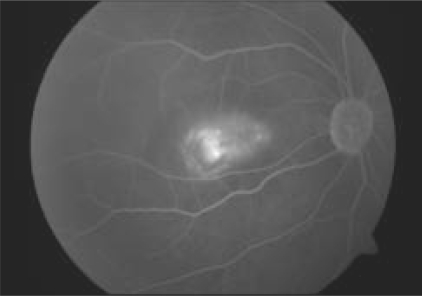
At 12 weeks, fundus fluorescein angiography reveals persisting leakage from the SRNVM

**Figure 4:**
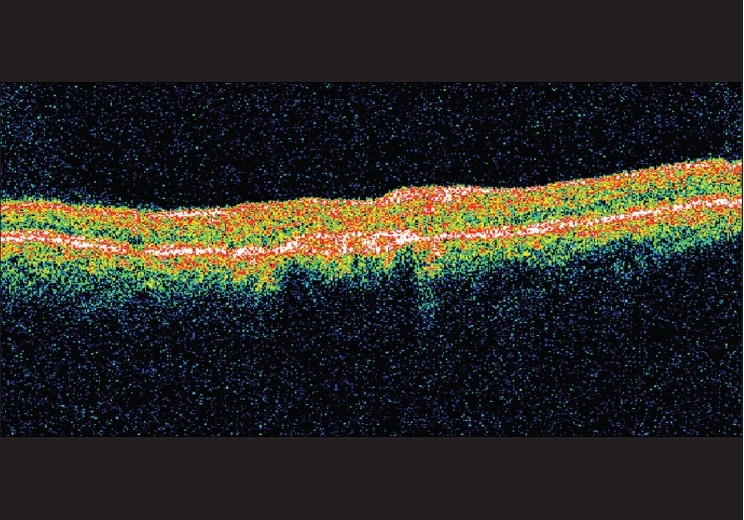
At 12 weeks, optical coherence tomography reveals increased retinal thickening overlying the SRNVM

**Figure 5:**
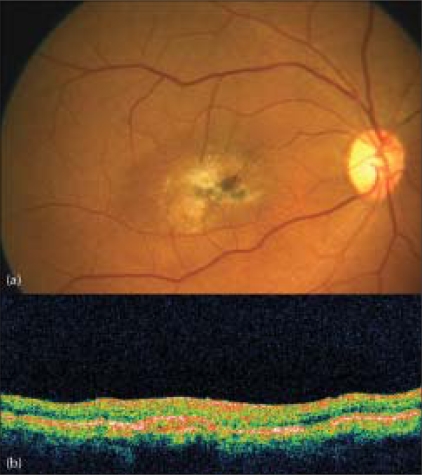
Color fundus picture (a) at the last follow-up reveals a completely regressed SRNVM. Optical coherence tomography (b) shows a high-reflective, scarred subretinal membrane

A 60-year-old gentleman presented with diminution of vision in the right eye for two months. Best corrected visual acuity (BCVA) was 20/600 in the right eye and 20/60 in the left eye. Fundus examination of both eyes revealed Type 2A IMT with SRNVM in the right eye.[7] Clinical findings were confirmed on fundus fluorescein angiography (FFA) and optical coherence tomography (OCT) [Figs. 1, 2]. The patient was informed about the various treatment options available and the ‘off label’ nature of bevacizumab. The patient underwent PDT followed by intravitreal bevacizumab (1.25 mg/0.05 ml), the next day. At 12 weeks follow-up, visual acuity in the right eye improved to 20/200. Clinically, SRNVM appeared to be regressing. The FFA showed minimal leakage [Fig. 3] and OCT revealed increased retinal thickness [Fig. 4]. Intravitreal bevacizumab (1.25 mg/0.05 ml) was repeated. At the last follow-up at 16 months, BCVA was maintained at 20/200 with a scarred SRNVM [Fig. 5].

PDT with verteporfin reportedly maintains baseline vision in such eyes.[4] Potter *et al.* reported an average of 2.4 PDT treatments for cessation of leakage.[4] Intravitreal bevacizumab has been reported to improve visual outcome and reduce leakage in these cases.[5] Bevacizumab would have a beneficial effect on SRNVM in IMT due to its location above the retinal pigment epithelium (RPE) and the presence of anastomotic retinal vascular connections which could facilitate the concentration of the dye in the SRNVM.[5] Although vascular endothelial growth factor (VEGF) inhibition alone could prevent neovascularization at an early developmental stage, once neovascular beds are established they are unlikely to regress with anti-VEGF therapy alone. At this stage, a combined approach using a non-thermal laser has been seen to be beneficial. Bevacizumab is a larger molecule (molecular weight ≈ 150 kD) with a slightly longer intravitreal half-life of 4.9 days as compared to three days for ranibizumab (molecular weight ≈ 50 kD). Also, bevacizumab is available at a fraction of the cost of ranibizumab. Hence, a combination of PDT with bevacizumab may in some way, score over a combination of PDT with ranibizumab in such eyes. In conclusion, combination therapy using PDT and bevacizumab appears to be a promising approach in the primary management of SRNVM secondary to IMT.
